# Neurotoxicology of alcohol: a bibliometric and science mapping analysis

**DOI:** 10.3389/fphar.2023.1209616

**Published:** 2023-08-01

**Authors:** Paulo Fernando Santos Mendes, Daiane Claydes Baia-da-Silva, Wallacy Watson Pereira Melo, Leonardo Oliveira Bittencourt, Renata Duarte Souza-Rodrigues, Luanna Melo Pereira Fernandes, Cristiane do Socorro Ferraz Maia, Rafael Rodrigues Lima

**Affiliations:** ^1^ Laboratory of Functional and Structural Biology, Institute of Biological Sciences, Federal University of Pará, Belém, Brazil; ^2^ Department of Morphology and Physiological Sciences, Center of Sciences Biological and Health, State University of Pará, Belém, Brazil

**Keywords:** alcohol abuse, central nervous system, alcohol, alcoholism, bibliometric

## Abstract

Alcohol consumption is common in many societies and has increased considerably, resulting in many socioeconomic and public health problems. In this sense, studies have been carried out in order to understand the mechanisms involved in alcohol consumption and related harmful effects. This study aimed to identify and map the knowledge and to perform bibliometric analysis of the neurotoxicology of alcohol based on the 100 most cited articles. A search was carried out in the Web of Science Core Collection database and information was extracted regarding the journal, authors, keywords, year of publication, number of citations, country and continent of the corresponding author. For each selected manuscript, the study design, alcohol exposure model, dose, period of exposure, and effect on the central nervous system and research hotspots were mapped. The journal with the highest number of publications was Alcoholism: Clinical and Experimental Research (n = 11 papers), the author who contributed the most was Crews FT (n = 8 papers), the studies had a total of 288 keywords and 75% of the publications were from the United States of America. The experimental studies evaluated the effects of prenatal and postnatal exposure and were conducted in rats and mice using doses ranging from 2.5 to 14 g/kg/day, with administration by subcutaneous, intraperitoneal, intragastric, or inhalation route or with free access through drinking bottles. Among the studies mapped, the oldest one (1989) aimed to understand the systemic damage and mechanisms of action involved, while the most recent focused on understanding the receptors and mechanisms involved in addiction, as well as genetic factors. Our results show the panorama of the most widespread scientific production in the scientific community on the neurotoxicology of ethanol, a high prevalence was observed in studies that addressed fetal alcohol syndrome and/or the effects of ethanol on neurodevelopment.

## 1 Introduction

Worldwide, the use of substances such as alcohol, tobacco, and illicit drugs has increased over time. Alcohol consumption is a cultural habit of many societies, and it is estimated that more than 2 billion people use this substance worldwide (WHO, 2019). In addition, its use is one of the main causes of long-term disability and causes 3 million deaths annually in the world (Tolomeo et al., 2021) and as a result, it has become a growing public health and socioeconomic concern (Bitew et al., 2020).

In the United States, excessive ethanol consumption is responsible for 88,000 deaths each year, and one in every 10 deaths of adults of working age (Kanny et al., 2018). In 2004, the National Institute on Alcohol Abuse and Alcoholism (NIAAA) defined excessive alcohol consumption (binge drinking), as consuming four or more drinks for women and five or more for men in a 2-h interval or consumption that leads to a blood alcohol concentration above 0.08 g/dL (Dejong et al., 2019; Currie et al., 2020). In addition to this pattern of consumption, the term “high-intensity consumption” was recently adopted in reference to consumption two or three times greater than excessive alcohol consumption; however there is not yet consensus on use of this term (Chung et al., 2018; Patrick and Azar, 2018).

The effects of ethanol are influenced by the pattern of consumption and the concentration of alcohol in the blood is dose-dependent, being proportional to the type and duration of exposure and determined by the speed at which alcohol is absorbed, distributed, metabolized, and excreted (Zakhari, 2006).

**FIGURE 1:**
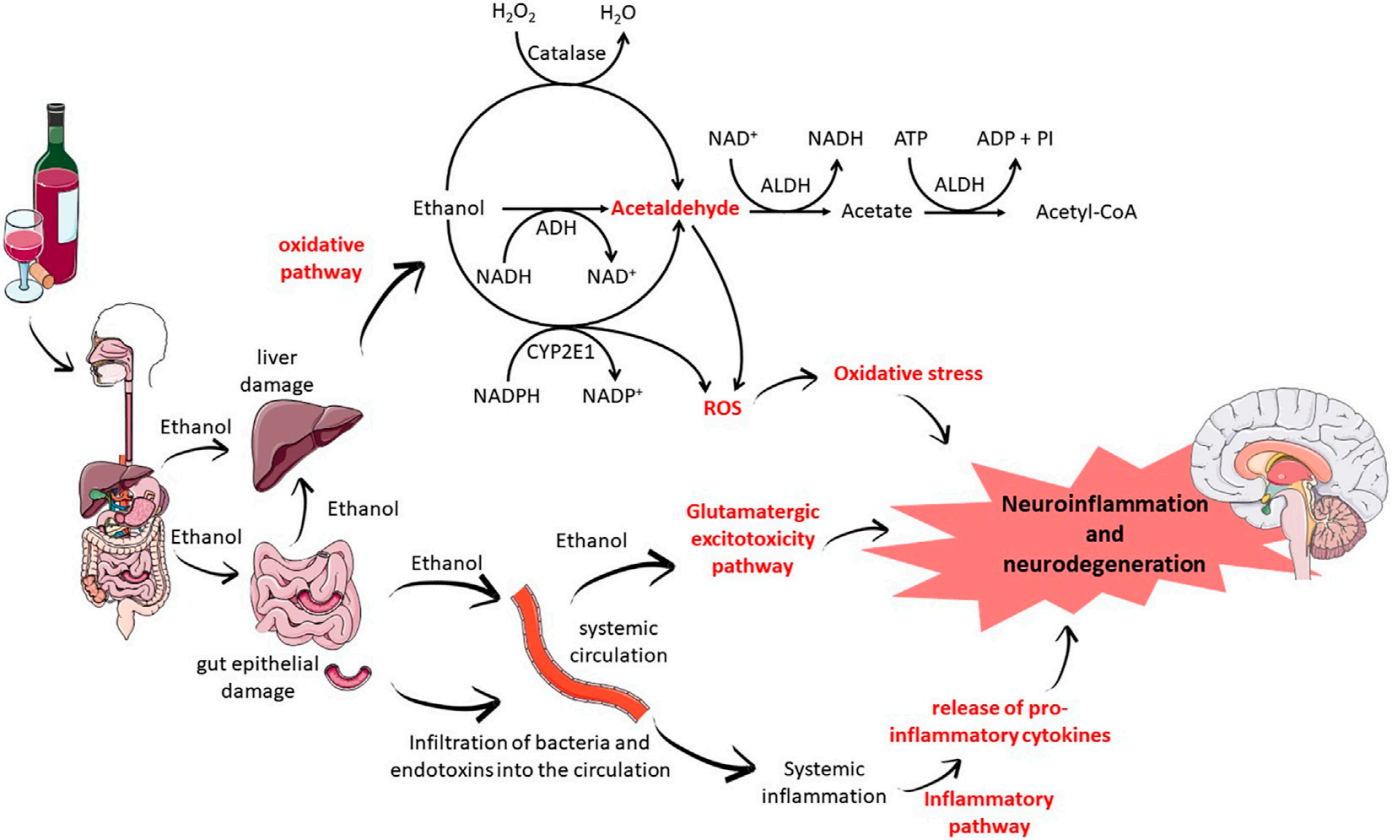
Mechanisms of alcohol-induced nervous system damage.

Regardless of the consumption pattern and the amount ingested, alcohol is essentially metabolized in the liver by enzymes such as alcohol dehydrogenase (ADH) and aldehyde dehydrogenase (ALDH). However, alcohol metabolism can also occur in other tissues, such as the brain, mediated by cytochrome p450 enzymes–especially CYP2E1—and catalase. The action of ADH and ALDH produces highly reactive and toxic intermediate metabolites in addition to reactive oxygen species (ROS), which can trigger changes in the redox state, triggering damage to various cells, tissues and organs (Zakhari, 2006; Fernandes et al., 2018a; Fernandes et al., 2018; Frazão et al., 2020; da Silva et al., 2018; Fernandes et al., 2015; Fernandes et al., 2018c). In Figure 1 we present the main mechanisms of damage triggered by ethanol to the nervous system (Figure 1).

The nervous system has been shown to be very sensitive to these oxidative changes, which can trigger neuroinflammatory conditions and thus trigger apoptotic processes (Salim, 2017). However, the mechanisms involved are not fully elucidated. Therefore, several groups have focused on understanding the mechanisms involved, including receptors, neurochemical changes, genetic factors and tissue response (Teixeira et al., 2014; Oliveira et al., 2015; Hamada and Lasek, 2020; Panula, 2020; García-Gutiérrez et al., 2022).

Given the socioeconomic impact of alcohol consumption, it is of great importance to know, understand and map these effects to search for alternatives for the prevention or treatment of damage to the nervous system induced by alcohol. Therefore, our objective was to identify, evaluate, and map the knowledge of the 100 most cited papers evaluating the neurotoxic effects of ethanol.

## 2 Materials and methods

### 2.1 Search strategy

To map the information produced about the neurotoxicology of alcohol, bibliometric analysis tools were used, as described in previous studies by our group (de Lima et al., 2022).

Data from this study were collected in December 2022 from the Web of Science Core Collection (WoS-CC) database, with no restrictions on language or year of publication, using a search strategy that used terms related to alcohol and neurotoxicity (Table 1).

**TABLE 1:** Search strategy.

Database	Search strategy
Web of Science- Core Colection	TS=(alcohol or EtOH or ethanol or “repeated ethanol exposure” or “binge drinking” or “alcohol intake” or “alcohol use disorder” or “drinking behavior” or “alcohol drinking habit” or “grain alcohol” or “ethyl alcohol” or “ethyl alcohol abuse neurologic syndromes” or “Ethanol-induced nervous system disorders” or “alcohol induced disorders nervous system” or “alcohol abuse” or “alcohol disorders” or “alcohol abuse” or “alcohol dependence” or “alcohol addiction” or “alcohol use” or “alcoholic beverage” or “beverage consumption” or “alcoholism” or “alcohol consumption” or “alcohol dependence” or “alcohol-consumption” or “alcohol-induced disorders” or “binge ethanol exposure” or “bingelike”) AND TS=(neurodegeneration or “induced neurodegeneration” or neurotoxicology or neurotoxicity or “neuron damage” or “neurotoxic effects” or “central nervous system” or “nervous system” or “nervous system disease” or “neurologic disorder” or “neurological disorder” or “nervous system disorder” or “nervous system poisoning” or “neurotoxic disorder” or “neurophysiological” or “neurogenetic processes” or “neurobiological bases” or “neuropharmacological” or “alcohol-related brain damage” or “alcohol-related neurodevelopmental disorder” or “apoptotic neurodegeneration” or “brain damage” or “brain development” or “brain lesions”).

### 2.2 Selection of studies

**FIGURE 2:**
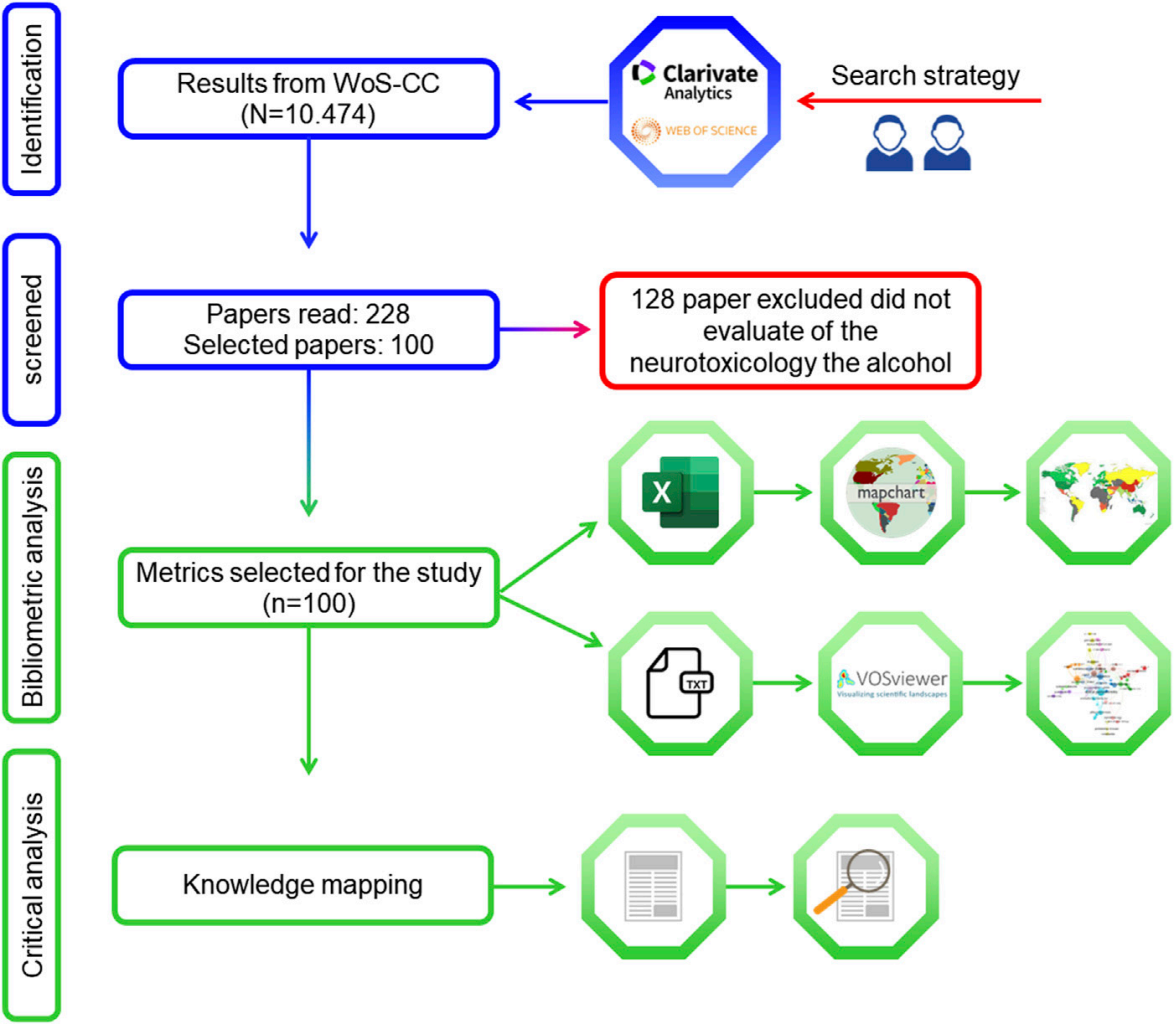
Flowchart of the literature search and article screening.

In WoS-CC, the results were organized in descending order of the number of citations. Two researchers (P.F.S.M. and D.C.B.-d.-S) independently carried out the selection of articles and data extraction after reading the title and abstract and then the full text, and differences of opinion were resolved by a third examiner (Nascimento et al., 2022; Corôa et al., 2023; de Sousa Né et al., 2023). The inclusion criteria were that the publications selected had to be articles that addressed the neurotoxicology of alcohol exposure. Editorials, comments, letters, and conference papers were excluded (Figure 2) (de Sousa Né et al., 2023).

### 2.3 Data extraction of bibliometric parameters

After selecting the 100 most cited articles, the following bibliometric parameters were collected: journal, authors, article title, keywords, number of citations, citation density, year of publication, DOI/URL, and country and continent of the corresponding author.

The rank was established based on decreasing order of the number of citations in the WoS-CC, and in the case of articles with identical numbers of citations, the citation density was used. The Scopus and Google Scholar databases were used for the purposes of comparison with the number of WoS-CC citations, since each database has unique criteria for quantification and data recording (Kulkarni et al., 2009).

### 2.4 Data analysis

The extracted data were exported to the software Visualization of Similarities Viewer (VOSviewer) version 1.6.16 (Centre for Science and Technology Studies, University of Leiden, the Netherlands) for analysis and construction of the collaboration network regarding the author and co-authorship and occurrences of the authors’ keywords (van Eck and Waltman, 2010). To create the networks, the authors’ names and keywords of authors with at least one article were introduced into the software as a unit of analysis. This software organizes terms into closely related nodes (clusters), represented by different colors, where the number of clusters is determined proportionally to the resolution parameter. The descriptive analysis of the data was performed using spreadsheet editing software (Microsoft Excel 365) and the graphical representation of the countries was generated using the MapChart tool (mapchart.net/index.html) (Figure 2).

### 2.5 Content analysis

For knowledge mapping, the selected articles were read in full and information such as the study design, model of exposure to ethanol, damage mechanisms associated with ethanol, pharmacological receptors, dose, exposure pathway, period of exposure, and cells and regions affected by use were extracted (Figure 2).

For the study design classification, the Cochrane Collaboration glossary was adopted as a reference: studies were classified as literature review, systematic review, laboratory studies (*in vitro*, *in vivo*, and *ex vivo*), practice guideline, case control, cohort study, case report, case series, cross-sectional study, randomized clinical study, or non-randomized clinical study.

## 3 Results

### 3.1 Selected studies and bibliometric analysis

The WoS-CC search yielded a total of 10,474 results, of which 228 were read, and the 100 most cited were selected (Figure 2). The selected articles received a total of 37,743 citations; the most cited article was “Critical Periods of Vulnerability for the Developing Nervous System: Evidence from Humans and Animal Models” (1,998 citations) (Rice and Barone, 2000) and the least cited was “Selective impairment of hippocampal neurogenesis by chronic alcoholism: Protective effects of an antioxidant” (Herrera et al., 2003; 209 citations). The average number of citations per article was 377.63. For most articles, the number of citations in WoS-CC was lower than the number of citations in Google Scholar and Scopus. The mean citation density in WoS-CC was 37.55. The article with the lowest citation density (7.38) was “Ethanol and the Nervous-System” (Charness et al., 1989) and the one with the highest citation density (86.67) was “Critical Periods of Vulnerability for the Developing Nervous System: Evidence from Humans and Animal Models” (Rice and Barone, 2000) (Table 2).

**TABLE 2:** General data of the 100 selected articles about the effects of alcohol on the central nervous system.

R^1^	Author/Years	Article title/DOI or URL	Description	Number of citations		
				WoS-CC^b^ (citation density ^c^)	Scopus	Google scholar
1	Rice and Barone (2000)	Critical periods of vulnerability for the developing nervous system: Evidence from humans and animal models/10.2307/3454543	Evaluated the neurodevelopmental impairment associated with alcohol exposure	1998 (86.87)	2176	3226
2	Koob (1992)	Drugs of abuse—anatomy, pharmacology and function of reward pathways/10.1016/0165-6147 (92)90060-J	Evaluated reward and addiction pathways associated with alcohol exposure	1829 (59.00)	1907	2827
3	Dunwiddie and Masino (2001)	The role and regulation of adenosine in the central nervous system/10.1146/annurev.neuro.24.1.31	Evaluated the neuromodulatory effect of exposure to alcohol	1176 (53.45)	1263	1835
4	Ikonomidou et al. (2000)	Ethanol-induced apoptotic neurodegeneration and fetal alcohol syndrome/10.1126/ciência.283.5398	Evaluated fetal alcohol syndrome and apoptotic neurodegeneration associated with ethanol exposure	1081 (47.00)	1204	1612
5	Koob (2008)	A role for brain stress systems in addiction/10.1016/j.neuron. 2008.06.012	Evaluated reward and addiction pathways associated with alcohol exposure	692 (46.13)	754	1136
6	Horrocks and Yeo (1999)	Health benefits of docosahexaenoic acid (DHA)/10.1006/phrs. 1999.0495	Evaluated the neurodevelopmental impairment associated with alcohol exposure	685 (28.54)	771	1389
7	Boden and Fergusson (2011)	Alcohol and depression/10.1111/j.1360-0443.2010.03351.x	Evaluated depression associated with exposure to ethanol	650 (54.17)	682	1164
8	Hoyme et al. (2005)	A practical clinical approach to diagnosis of fetal alcohol spectrum disorders: Clarification of the 1996 Institute of Medicine Criteria/10.1542/peds. 2004-0259	Evaluated clinical approach in diagnosing fetal alcohol spectrum disorders	642 (35.67)	731	1133
9	Fiala et al. (2002)	Dendritic spine pathology: Cause or consequence of neurological disorders?/10.1016/S0165-0173 (02)00158-3	Evaluated neurological disorders and cognitive deficits associated with exposure to alcohol	615 (29.29)	660	970
10	Chudley et al. (2005)	Fetal alcohol spectrum disorder: Canadian guidelines for diagnosis/10.1503/cmaj.1040302	Evaluated fetal alcohol syndrome associated with alcohol exposure	608 (33.78)	688	1198
11	Clarren et al. (1997)	Incidence of fetal alcohol syndrome and prevalence of alcohol-related neurodevelopmental disorder/10.1002/(SICI)1096-9926 (199711)56:5 < 317:AID-TERA5>3.0.CO;2-U	Evaluated fetal alcohol syndrome associated with alcohol exposure	574 (22.08)	670	1126
12	Polich and Criado (2006)	Neuropsychology and neuropharmacology of P3a and P3b/10.1016/j.ijpsycho. 2005.12.012	Evaluated the effect of alcohol on brain potential related to events	524 (30.82)	555	841
13	Nordmann et al. (1992)	Implication of free-radical mechanisms in ethanol-induced cellular injury/10.1016/0891-5849 (92)90030-K	Evaluated oxidative damage associated with alcohol exposure	520 (16.77)	556	813
14	Liou et al. (1997)	Environmental risk factors and Parkinson’s disease: A case-control study in Taiwan/10.1212/WNL.48.6.1583	Evaluated the risk of Parkinson’s disease associated with exposure to alcohol	504 (19.38)	568	838
15	Courtney and Polich (2009)	Binge drinking in young adults: data, definitions, and determinants/10.1037/a0014414	Evaluated the epidemiology, definitions and determinants of excessive alcohol consumption in young adults	476 (34.00)	495	872
16	McBride and Li (1998)	Animal models of alcoholism: Neurobiology of high alcohol-drinking behavior in rodents/10.1615/critrevneurobiol.v12.i4.40	Evaluated neurobiological and behavioral mechanisms associated with the abusive use of ethanol	476 (19.04)	502	674
17	Eckardt et al. (1998)	Effects of moderate alcohol consumption on the central nervous system/10.1111/j.1530-0277.1998.tb03695.x	Evaluated the effects of alcohol consumption on the central nervous system	472 (18.88)	510	818
18	McKinney et al. (2007)	Posterior reversible encephalopathy syndrome: Incidence of atypical regions of involvement and imaging findings/10.2214/AJR.07.2024	Evaluated clinical manifestations and regions involved in reversible posterior encephalopathy syndrome associated with alcohol consumption	455 (28.44)	514	802
19	Pich et al. (1995)	Increase of extracellular corticotropin-releasing factor-like immunoreactivity levels in the amygdala of awake rats during restraint stress and ethanol withdrawal as measured by microdialysis/10.1523/JNEUROSCI.15-08-05439.1995	Evaluated the corticotropin-releasing factor in the anxiogenic effects stimulated by ethanol abstinence	451 (16.11)	504	623
20	Crews and Boettiger (2009)	Impulsivity, frontal lobes and risk for addiction/10.1016/j.pbb. 2009.04.018	Evaluated cortical changes associated with alcohol consumption	446 (31.86)	475	830
21	May and Gossage (2001)	Estimating the prevalence of fetal alcohol syndrome—A summary/https://pubmed.ncbi.nlm.nih.gov/11810953/	Evaluated the prevalence of fetal alcohol syndrome in the United States	442 (20.09)	493	888
22	Bonthius and West (1990)	Alcohol-induced neuronal loss in developing rats—increased brain-damage with binge exposure/10.1111/j.1530-0277.1990.tb00455.x	Evaluated the lowest daily dose of alcohol capable of inducing neuronal loss, as well as the regions most sensitive to damage	434 (13.15)	460	630
23	Mehta et al. (2013)	Excitotoxicity: Bridge to various triggers in neurodegenerative disorders/10.1016/j.ejphar. 2012.10.032	Reviewed the role of calcium, mitochondrial dysfunction, reactive oxygen species, nitric oxide, chloride homeostasis, and eicosanoid pathways in the stimulation and maintenance of neuronal excitation	427 (42.70)	452	663
24	Riley et al. (2011)	Fetal Alcohol Spectrum Disorders: An Overview/10.1007/s11065-011-9166-x	Evaluated changes in brain development associated with fetal alcohol syndrome	422 (35.17)	456	760
25	De Bellis et al. (2000)	Hippocampal volume in adolescent-onset alcohol use disorders/10.1176/appi.ajp.157.5.737	Evaluated the volume of the hippocampus of adolescents and adults with disorders due to alcohol use with those of healthy individuals	409 (17.78)	473	837
26	Fadda and Rossetti (1998)	Chronic ethanol consumption: From neuroadaptation to neurodegeneration/10.1016/S0301-0082 (98)00032-X	Evaluated neurobiological changes induced by alcohol as well as the neuropathological consequences of abuse for cognitive functions and aerial structures	407 (16.28)	456	699
27	Kril et al. (1997)	The cerebral cortex is damaged in chronic alcoholics/10.1016/S0306-4522 (97)00083-3	Evaluated neurodegeneration of the cerebral cortex of alcoholics	387 (14.88)	413	617
28	Crews and Nixon (2009)	Mechanisms of Neurodegeneration and Regeneration in Alcoholism/10.1093/alcalc/agn079	Evaluated neuronal death and loss of neurogenesis induced by alcohol, as well as the effects induced by abstinence	386 (27.57)	411	611
29	Grobin et al. (1998)	The role of GABA(A) receptors in the acute and chronic effects of ethanol/10.1007/s002130050685	Evaluated the interactions of alcohol with GABAA receptors and their association with the acute actions, tolerance, dependence and self-administration of ethanol	383 (15.32)	412	593
30	Crews et al. (2000)	Binge ethanol consumption causes differential brain damage in young adolescent rats compared with adult rats/10.1111/j.1530-0277.2000.tb01973.x	Evaluated the effects and brain damage induced by excessive alcohol consumption	382 (16.61)	411	629
31	Deitrich et al. (1989)	Mechanism of action of ethanol-initial central-nervous-system actions/https://pharmrev.aspetjournals.org/content/41/4/489	Evaluated the mechanisms of action of alcohol in the central nervous system	381 (11.21)	402	584
32	Moselhy et al. (2001)	Frontal lobe changes in alcoholism: A review of the literature/10.1093/alcalc/36.5.357	Evaluated the neurophysiological, morphological and neuropsychological effects of alcohol on the central nervous system	378 (17.18)	425	734
33	Alfonso-Loeches et al. (2010)	Pivotal Role of TLR4 Receptors in Alcohol-Induced Neuroinflammation and Brain Damage/10.1523/JNEUROSCI.0976-10.2010	Evaluated Toll-like receptors in ethanol-induced glial activation, as well as brain damage resulting from this activation	370 (28.46)	417	552
34	Heinz et al. (2000)	A relationship between serotonin transporter genotype and *in vivo* protein expression and alcohol neurotoxicity/10.1016/S0006-3223 (99)00171-7	Evaluated the genetic variation of the serotonin transporter (5-HTT) and its association with susceptibility to the neurotoxic effects of chronic excessive alcohol consumption	366 (15.91)	410	539
35	May et al. (2018)	Prevalence of fetal alcohol spectrum disorders in 4 US communities/10.1001/jama. 2017.21896	Evaluated the prevalence of fetal alcohol spectrum disorders and alcohol-related neurodevelopmental disorders in the US.	353 (70.60)	378	553
36	He and Crews (2008)	Increased MCP-1 and microglia in various regions of the human alcoholic brain/10.1016/j.expneurol. 2007.11.017	Evaluated microglial activation by pro-inflammatory cytokines and their association with damage induced by alcohol abuse	352 (23.47)	380	483
37	Qin et al. (2008)	Increased systemic and brain cytokine production and neuroinflammation by endotoxin following ethanol treatment/10.1186/1742-2094-5-10	Evaluated gene expression and protein synthesis of cytokines, oxidative enzymes, microglial activation and inhibition of neurogenesis induced by lipopolysaccharide and whether alcohol abuse potentiates its effects	347 (23.13)	397	532
38	Nixon and Crews (2002)	Binge ethanol exposure decreases neurogenesis in adult rat hippocampus/10.1046/j.1471-4159.2002.01214.x	Evaluated the effect of alcohol exposure on proliferation and survival of neural progenitor cells	345 (16.43)	380	541
39	Sullivan et al. (2000)	Pattern of motor and cognitive deficits in detoxified alcoholic men/10.1111/j.1530-0277.2000.tb02032.x	Evaluated executive functions, visuospatial skills, gait, and balance of individuals detoxified by alcohol for a month with healthy individuals	332 (14.43)	361	546
40	Squeglia et al. (2009)	The influence of substance use on adolescent brain development/10.1177/155005940904000110	Evaluated studies on neurocognition, brain structure and function in adolescents exposed to alcohol and marijuana	321 (22.93)	338	669
41	Weiss et al. (2006)	Compulsive drug-seeking behavior and relapse—Neuroadaptation, stress, and conditioning factors/10.1111/j.1749-6632.2001.tb03556.x	Evaluated the neuroadaptive changes that oppose the acute reinforcement actions of drugs of abuse, as well as the onset of anxiety, dysphoria, and depression during abstinence	320 (14.55)	352	525
42	Casey et al. (2011)	Braking and accelerating of the adolescent brain/10.1111/j.1532-7795.2010.00712.x	Evaluated the mechanisms involved in increased sensitivity, motivational cues, and cognitive control in adolescence and their association with alcohol and drug abuse	310 (25.83)	349	692
43	Sullivan and Pfefferbaum (2005)	Neurocircuitry in alcoholism: A substrate of disruption and repair/10.1007/s00213-005-2267-6	Evaluated by neuroimaging the alterations in the structure, physiology, and function of the neural systems induced by excessive alcohol consumption	310 (17.22)	345	528
44	Crews et al. (2006)	Cytokines and alcohol/10.1111/j.1530-0277.2006.00084.x	Evaluated cytokine production in response to alcohol abuse	307 (18.06)	330	470
45	Lewohl et al. (2000)	Gene expression in human alcoholism: Microarray analysis of frontal cortex/10.1111/j.1530-0277.2000.tb01993.x	Evaluated changes in brain gene expression responsible for tolerance, dependence, and neurotoxicity induced by alcohol abuse	305 (13.26)	329	430
46	Kumar et al. (2009)	The role of GABA(A) receptors in the acute and chronic effects of ethanol: A decade of progress/10.1007/s00213-009-1562-z	Evaluated mechanisms of action of ethanol in the nervous system mediated by the GABAA receptor	303 (21.64)	335	492
47	Tsai et al. (1995)	The glutamatergic basis of human alcoholism/10.1176/ajp.152.3.332	Evaluated the effects of alcohol on glutamatergic transmission and its possible association with alcohol-related problems	295 (10.54)	324	533
48	Hoyme et al. (2016)	Updated clinical guidelines for diagnosing fetal alcohol spectrum disorders/10.1542/peds. 2015-4256	Guidelines for the management of children with potential fetal alcohol spectrum disorders	291 (41.57)	359	560
49	Chanraud et al. (2007)	Brain morphometry and cognitive performance in detoxified alcohol-dependents with preserved psychosocial functioning/10.1038/sj.npp.1301219	Evaluated the extent of brain structure damage and cognitive deficits in alcohol-dependent men	289 (18.06)	317	489
50	Grace (2000)	The tonic/phasic model of dopamine system regulation and its implications for understanding alcohol and psychostimulant craving/10.1080/09652140050111690	Evaluated the tonic versus phasic relationship in the dopaminergic system and its association with dependence, abstinence, and continued desire for drug use	288 (12.52)	319	512
51	Diamond and Gordon (1997)	Cellular and molecular neuroscience of alcoholism/10.1152/physrev. 1997.77.1.1	Evaluated changes in the transduction of signals activated by neurotransmitter hormones that lead to changes in cell function and gene expression in alcoholism	287 (11.04)	316	458
52	Astley and Clarren (2000)	Diagnosing the full spectrum of fetal alcohol-exposed individuals: Introducing the 4-Digit Diagnostic Code/10.1093/alcalc/35.4.400	Evaluated a new diagnostic method for fetal alcohol spectrum syndrome and compared it with the gestalt method	285 (12.39)	347	617
53	Harding et al. (2000)	Degeneration of anterior thalamic nuclei differentiates alcoholics with amnesia/10.1093/brain/123.1.141	Evaluated degeneration of amnesic and non-amnesic alcoholic anterior thalamic nuclei	281 (12.22)	322	462
54	Windle et al. (2008)	Transitions into underage and problem drinking: Developmental processes and mechanisms between 10 and 15 years of age/10.1542/peds. 2007-2243C	Evaluated the impairments in neurodevelopment resulting from exposure to alcohol in the age group of 10–15 years	279 (18.60)	291	459
55	LeMarquand et al. (1994)	Serotonin and alcohol intake, abuse, and dependence—clinical-evidence/10.1016/0006-3223 (94)90630-0	Evaluated the effects and alterations triggered by alcohol consumption in the serotonergic system, in normal and dependent patients	279 (9.62)	208	422
56	Berman and Hannigan (2000)	Effects of prenatal alcohol exposure on the hippocampus: Spatial behavior, electrophysiology, and neuroanatomy/10.1002/(SICI)1098-1063 (2000)10:1 < 94:AID-HIPO11 > 3.0.CO;2-T	Evaluated the teratogenic effects of prenatal alcohol exposure on the hippocampus	274 (11.91)	291	416
57	Bava and Tapert (2010)	Adolescent brain development and the risk for alcohol and other drug problems/10.1007/s11065-010-9146-6	Evaluated neurodevelopment in adolescence and the changes and mechanisms involved as a result of alcohol consumption	272 (20.92)	300	605
58	Zeigler et al. (2005)	The neurocognitive effects of alcohol on adolescents and college students/10.1016/j.ypmed. 2004.04.044	Evaluated neurodevelopment in adolescence and the changes and mechanisms involved as a result of alcohol consumption	272 (15.11)	299	652
59	Harper (1998)	The neuropathology of alcohol-specific brain damage, or does alcohol damage the brain?/10.1097/00005072-199802000-00001	Evaluated neuropathological changes related to excessive and prolonged alcohol use	272 (10.88)	325	474
60	Roberto et al. (2003)	Ethanol increases GABAergic transmission at both pre- and postsynaptic sites in rat central amygdala neurons/10.1073/pnas.0437926100	Evaluated the ethanol–GABA interaction in the central nucleus of the amygdala and its relationship with the potentiating effects of ethanol	270 (13.50)	286	400
61	Caine et al. (1997)	Operational criteria for the classification of chronic alcoholics: Identification of Wernicke’s encephalopathy/10.1136/jnnp.62.1.51	Evaluated operational criteria for the diagnosis of Wernicke’s encephalopathy	270 (10.38)	307	504
62	Roberts et al. (1996)	Intra-amygdala muscimol decreases operant ethanol self-administration in dependent rats/10.1111/j.1530-0277.1996.tb01125.x	Evaluated a model for evaluating reinforcement-related mechanisms and neurotransmitter pathways involved in alcohol reward	268 (9.93)	288	335
63	Pascual et al. (2007)	Intermittent ethanol exposure induces inflammatory brain damage and causes long-term behavioural alterations in adolescent rats/10.1111/j.1460-9568.2006.05298.x	Evaluated the inflammatory pathways involved in alcohol-induced brain damage	265 (16.56)	292	414
64	VanDoren et al. (2000)	Neuroactive steroid 3 alpha-hydroxy-5 alpha-pregnan-20-one modulates electrophysiological and behavioral actions of ethanol/10.1523/JNEUROSCI.20-05-01982.2000	Evaluated the association between increased levels of the neurosteroid allopregnanolone and the hypnotic effect of alcohol	261 (11.35)	292	333
65	De Bellis (2002)	Developmental traumatology: A contributory mechanism for alcohol and substance use disorders/10.1016/S0306-4530 (01)00042-7	Evaluated the association between childhood trauma and increased risk of problems with alcohol and other drug use	260 (12.38)	298	516
66	Goodlett et al. (2005)	Alcohol teratogenesis: Mechanisms of damage and strategies for intervention/10.1177/15353702-0323006-07	Evaluated the mechanisms involved in the development of the neuroteratogenic effects of alcohol	259 (14.39)	293	466
67	Romero et al. (1998)	Lipid peroxidation products and antioxidants in human disease/10.2307/3433990	Evaluated the association between increased lipid peroxidation products and their influence on antioxidant levels	256 (10.24)	315	547
68	Fernandez-Lizarbe et al. (2009)	Critical role of TLR4 response in the activation of microglia induced by ethanol/10.4049/jimmunol.0803590	Evaluated TLR4 receptors in microglial activation induced by alcohol consumption	254 (18.14)	282	363
69	Roberts et al. (2000)	mu-opioid receptor knockout mice do not self-administer alcohol/https://jpet.aspetjournals.org/content/293/3/1002	Evaluated the effect of μ-opioid receptors on the alcohol reinforcement system and its possible therapeutic target	252 (10.96)	271	373
70	Charness et al. (1989)	Ethanol and the nervous-system/10.1056/NEJM198908173210706	Evaluated the effects of alcohol on the nervous system and its association with consumption patterns	251 (7.38)	279	462
71	de Fonseca et al. (2005)	The endocannabinoid system: Physiology and pharmacology/10.1093/alcalc/agh110	Evaluated the application of cannabinoid receptor antagonists as a therapeutic alternative to alcohol addiction	248 (13.78)	287	503
72	Crews et al. (1996)	Effects of ethanol on ion channels/10.1016/s0074-7742 (08)60670-4	Evaluated the role of ion channels in the mechanisms of alcohol toxicity	248 (9.19)	261	344
73	Hommer et al. (2001)	Evidence for a gender-related effect of alcoholism on brain volumes/10.1176/appi.ajp.158.2.198	Evaluated sensitivity to alcohol-induced neurotoxicity between genders	245 (11.14)	283	418
74	Thompson et al. (2009)	Prenatal exposure to drugs: Effects on brain development and implications for policy and education/10.1038/nrn2598	Evaluated neurodevelopmental changes triggered by prenatal alcohol exposure	243 (17.36)	264	400
75	Townshend and Duka (2005)	Binge drinking, cognitive performance and mood in a population of young social drinkers/10.1097/01.ALC.0000156453.05028.F5	Evaluated the cognitive performance of compulsive drinkers and compared them to non-compulsive drinkers	243 (13.50)	256	414
76	Porjesz et al. (2005)	The utility of neurophysiological markers in the study of alcoholism/10.1016/j.clinph. 2004.12.016	Evaluated the association between changes in electroneurophysiological markers and predisposition to develop alcohol-related problems	242 (13.44)	264	338
77	Olney et al. (2002)	Ethanol-induced apoptotic neurodegeneration in the developing C57BL/6 mouse brain/10.1016/S0165-3806 (02)00279-1	Evaluated ethanol-induced apoptotic neurodegeneration in mice during synaptogenesis	240 (11.43)	258	324
78	Medina et al. (2008)	Prefrontal cortex volumes in adolescents with alcohol use disorders: Unique gender effects/10.1111/j.1530-0277.2007.00602.x	Evaluated the association between alcohol abuse in adolescence and reduced volume of the prefrontal cortex	237 (15.80)	260	387
79	Riley et al. (1995)	Abnormalities of the corpus-callosum in children prenatally exposed to alcohol/10.1111/j.1530-0277.1995.tb01600.x	Evaluated the corpus callosum of children exposed to high doses of alcohol in the prenatal period	237 (8.46)	279	418
80	Guerri et al. (2009)	Foetal alcohol spectrum disorders and alterations in brain and behaviour/10.1093/alcalc/agn105	Evaluated the effects of ethanol on the nervous system and its association with cognitive, behavioral and psychopathological deficits	236 (16.86)	261	419
81	Harper (2009)	The neuropathology of alcohol-related brain damage//10.1093/alcalc/agn102	Evaluated the human brain image bank to assess changes induced by alcohol abuse	236 (16.86)	272	459
82	Harper and Matsumoto (2005)	Ethanol and brain damage/10.1016/j.coph. 2004.06.011	Evaluated the mechanisms underlying brain damage induced by alcohol exposure	236 (13.11)	278	470
83	Brower (2003)	Insomnia, alcoholism and relapse/10.1016/S1087-0792 (03)90005-0	Assessed insomnia in alcohol abstinence and its association with relapse	236 (11.80)	273	389
84	Sayette (1993)	An appraisal-disruption model of alcohols effects on stress responses in social drinkers/10.1037/0033-2909.114.3.459	Evaluated the effects of alcohol on stress responses among social drinkers	235 (7.83)	251	455
85	Popova et al. (2016)	Comorbidity of fetal alcohol spectrum disorder: a systematic review and meta-analysis/10.1016/S0140-6736 (15)01345-8	Evaluated the comorbidities associated with fetal alcohol aspect disorder	234 (33.43)	260	465
86	Wozniak et al. (2004)	Apoptotic neuro degeneration induced by ethanol in neonatal mice is associated with profound learning/memory deficits in juveniles followed by progressive functional recovery in adults/10.1016/j.nbd. 2004.08.006	Evaluated neuronal apoptotic death of hippocampal circuit structures and its association with reduced spatial memory capacity	232 (12.21)	247	320
87	Mumenthaler et al. (1999)	Gender differences in moderate drinking effects/https://www-ncbi-nlm-nih.ez3.periodicos.capes.gov.br/pmc/articles/PMC6761697/	Evaluated the difference in the effects of alcohol between genders	231 (9.63)	257	450
88	Harris et al. (1995)	Mutant mice lacking the gamma-isoform of protein-kinase-c show decreased behavioral actions of ethanol and altered function of gamma-aminobutyrate type-a receptors/10.1073/pnas.92.9.3658	Evaluated calcium/phospholipid-dependent protein kinase and its role in the sensitivity of type A gamma-aminobutyrate receptors to ethanol	225 (8.04)	226	314
89	Martin et al. (2003)	The role of thiamine deficiency in alcoholic brain disease/https://www-ncbi-nlm-nih.ez3.periodicos.capes.gov.br/pmc/articles/PMC6668887/	Evaluated the nutritional deficiency of thiamine resulting from chronic alcohol consumption and its association with alcohol-induced brain damage	223 (11.15)	262	469
90	Guerri and Pascual (2010)	Mechanisms involved in the neurotoxic, cognitive, and neurobehavioral effects of alcohol consumption during adolescence/10.1016/j.alcohol. 2009.10.003	Evaluated the mechanism of neurotoxic, cognitive, and neurobehavioral effects induced by alcohol consumption during adolescence	222 (17.08)	241	430
91	Haorah et al. (2008)	Mechanism of alcohol-induced oxidative stress and neuronal injury/10.1016/j.freeradbiomed. 2008.08.030	Evaluated neurocognitive deficits, neuronal injury, and neurodegeneration associated with the progression of neuroinflammation triggered by oxidative damage to mitochondria and cellular proteins	222 (14.80)	257	338
92	Bookstein et al. (2002)	Corpus callosum shape and neuropsychological deficits in adult males with heavy fetal alcohol exposure/10.1006/nimg. 2001.0977	Evaluated neuropsychological deficits associated with changes in the shape of the corpus callosum resulting from fetal alcohol exposure	219 (10.43)	248	362
93	Begleiter and Porjesz (1999)	What is inherited in the predisposition toward alcoholism? A proposed model/10.1111/j.1530-0277.1999.tb04269.x	Evaluated the association between predisposition to alcoholism and hereditary hyperexcitability of the nervous system	215 (8.96)	228	325
94	Livy et al. (2003)	Fetal alcohol exposure and temporal vulnerability: Effects of binge-like alcohol exposure on the developing rat hippocampus/10.1016/S0892-0362 (03)00030-8	Evaluated fetal alcohol exposure and changes in the hippocampus, as well as changes in the learning and memory process	214 (10.70)	226	312
95	Costa et al. (2004)	Developmental neuropathology of environmental agents/10.1146/annurev.pharmtox.44.101802.121424	Evaluated morphological changes associated with alcohol consumption	213 (11.21)	256	359
96	Olney et al. (2006)	Drug-induced apoptotic neurodegeneration in the developing brain/10.1111/j.1750-3639.2002.tb00467.x	Evaluated the mechanisms of induction of alcohol-induced apoptotic neurodegeneration	213 (10.24)	244	343
97	Nagel et al. (2005)	Reduced hippocampal volume among adolescents with alcohol use disorders without psychiatric comorbidity/10.1016/j.pscychresns. 2005.05.008	Evaluated the reduction of hippocampal volume in adolescents with alcohol use disorders	211 (11.72)	240	364
98	Maier and West (2001)	Drinking patterns and alcohol-related birth defects/www-ncbi-nlm-nih.ez3.periodicos.capes.gov.br/pmc/articles/PMC6707176/pdf/arcr-25-3-168.pdf	Evaluated fetal alcohol exposure during and its association with cognitive and behavioral deficits	211 (9.59)	249	440
99	Rangaswamy et al. (2002)	Beta power in the EEG of alcoholics/10.1016/S0006-3223 (02)01362-8	Evaluated the beta potency in the electroencephalogram at rest to assess excitation-inhibition imbalance in the central nervous system of alcoholics	209 (9.95)	246	395
100	Herrera et al. (2003)	Selective impairment of hippocampal neurogenesis by chronic alcoholism: Protective effects of an antioxidant/10.1073/pnas.1230907100	Evaluated neurogenesis in the adult hippocampus and the impairment associated with oxidative damage induced by alcohol consumption	204 (10.20)	219	321

^a^ R = rank.

^b^ WoS-CC, web of science core collection.

^c^ Citation density = Mean based on the ratio of the number of citations and the period since the year of publication up to December 2022.

#### 3.1.1 Period of publication

**FIGURE 3:**
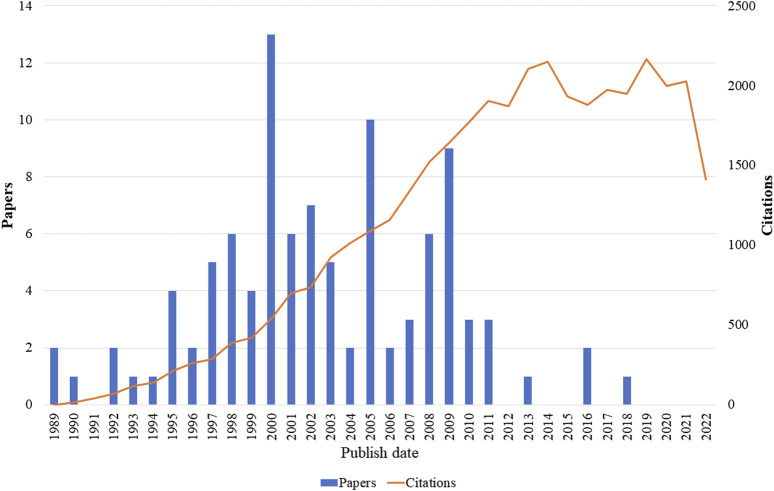
Publications and number of citations each year of the top 100 articles related to use of alcohol and central nervous system effects.

The two oldest articles were published in 1989 and the most recent in 2018 (Figure 3). The most recent article was “Prevalence of Fetal Alcohol Spectrum Disorders in 4 US Communities” (May et al., 2018) which had the objective of estimating the prevalence of fetal alcohol spectrum disorders and received 353 citations, with a citation density of 70.60. The oldest articles were “Ethanol and the nervous system” (Charness et al., 1989) and “Mechanism of Action of Ethanol: initial Central Nervous System Actions” (Deitrich et al., 1989) both of which proposed to evaluate the effects of alcohol and/or its metabolites on the nervous system, with the first receiving 251 citations and 7.38 citations per year while the second received 381 citations and an average of 11.21 citations per year.

The selected articles were published between the years 1989 and 2018; 2% of the articles were published in 1989, 25% between 1990 and 1999, 63% between 2000 and 2009, and 10% between 2010 and 2018. The year 2000 was the period with the highest number of publications selected in this study (n = 13) and the year 2005 had the second highest number of publications (n = 10). Regarding the number of citations, there was annual growth until the year 2011, when it reached 1,902. In 2012, there was a slight reduction in the number of citations, but in 2013 and 2014 the growth in the number of citations recovered, reaching 2,149 in 2014. In 2015, there was a reduction that remained stable until 2018. In 2019, the number of citations grew again, reaching 2,165, the maximum reached in the evaluated period (Figure 3).

Regarding the number of citations, there was annual growth until the year 2011, when it reached 1,902. In 2012, there was a slight reduction in the number of citations, but in 2013 and 2014 the growth in the number of citations recovered, reaching 2,149 in 2014. In 2015, there was a reduction that remained stable until 2018. In 2019, the number of citations grew again, reaching 2,165, the maximum reached in the evaluated period (Figure 3).

#### 3.1.2 Journal of publication

**FIGURE 4:**
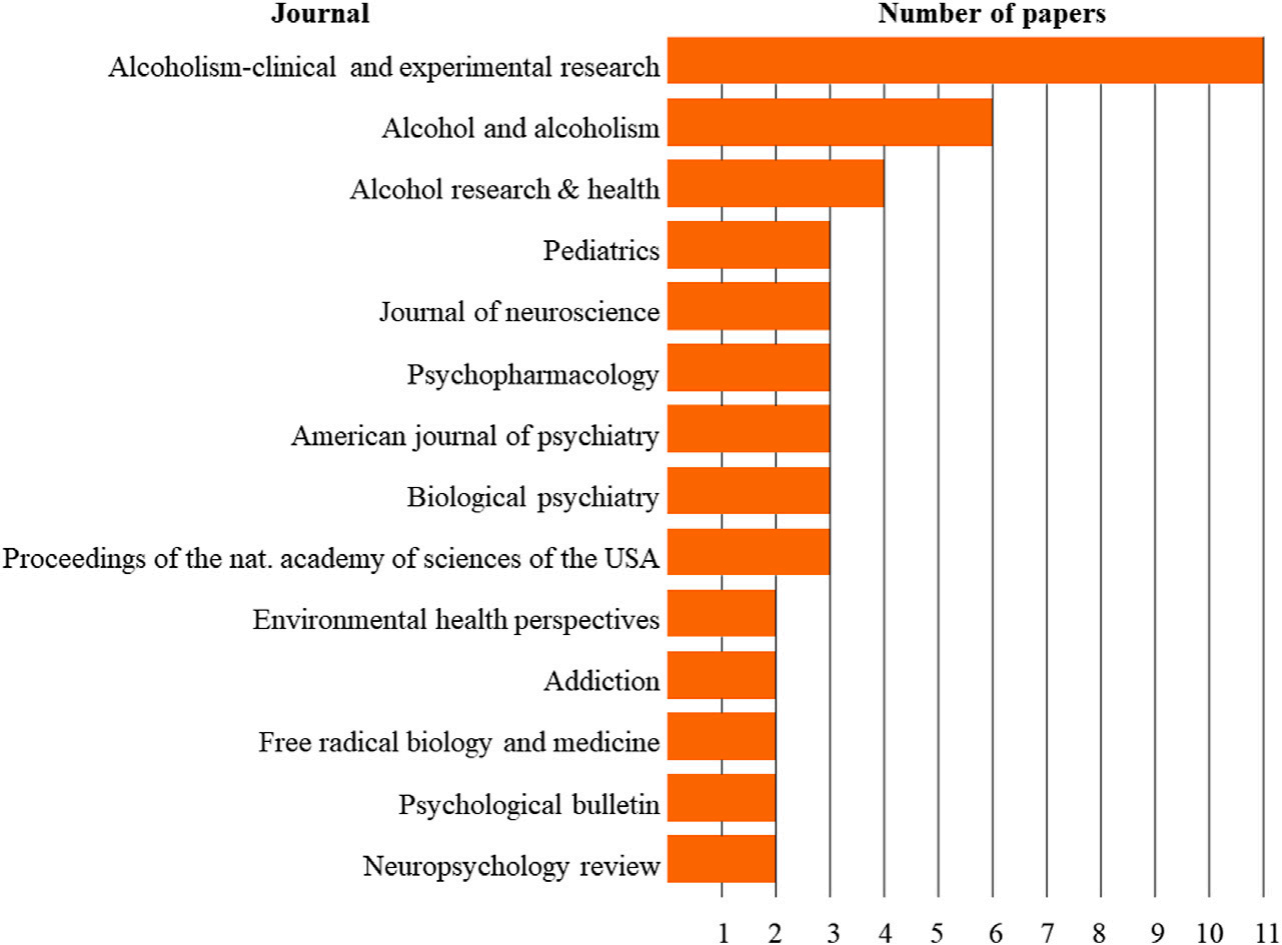
Journals with at least two published articles.

The articles were published in 65 journals, of which *Alcoholism: Clinical and Experimental Research* was the one with the most papers published (n = 11), followed by *Alcohol and Alcoholism* (n = 6) and *Alcohol Research & Health* (n = 4). Most of the journals contributed only one article (78.46%), 7.69% contributed two articles each, and 9.23% contributed three articles each (Figure 4).

#### 3.1.3 Contributing authors

**FIGURE 5:**
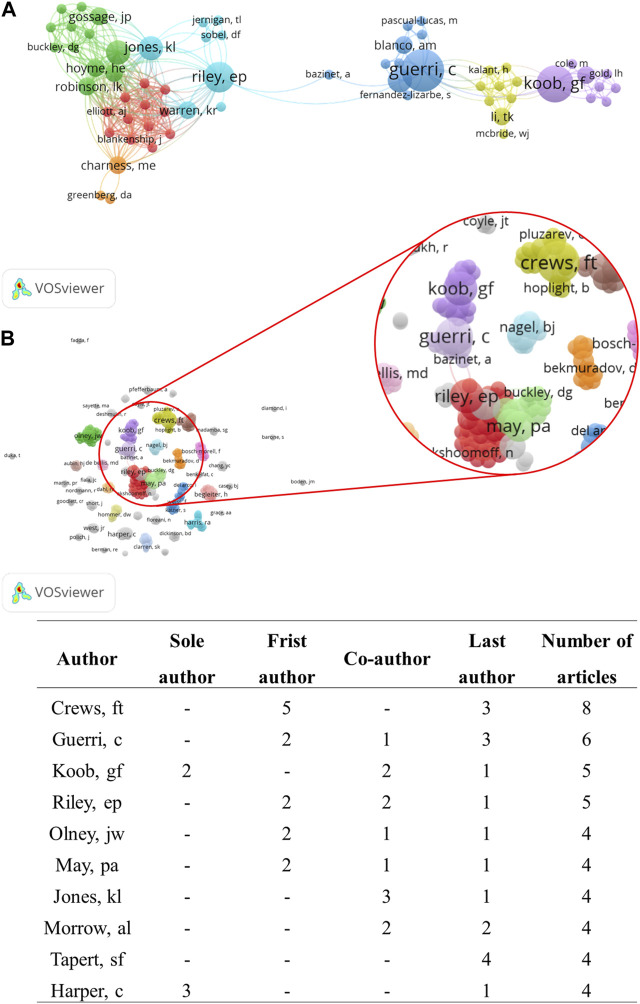
Network visualization of co-authorship: **(A)** The largest set of connected authors (n = 72 authors); **(B)** All authors with at least one papers (n = 371authors), showing the authors with the largest number of articles published about the neurotoxicology of alcohol. The node size represents the number of documents: the larger the node, the greater the number of articles. Clusters are represented by different colors and the lines indicate co-citation links between authors.

A total of 371 authors contributed to the selected papers. Crews FT, Guerri C, Koob GF, and Riley EP were the authors with the highest number of publications (n = 8, n = 6, n = 5, and n = 5, respectively). The most cited authors were Koob GF and Crews FT, who received 3,513 and 2,813 citations, respectively. Crews FT was first author on five articles and last author on three articles, while Koob GF was sole author on two articles, co-author on two articles and last author on one article. (Figure 5).

#### 3.1.4 Keywords

**FIGURE 6:**
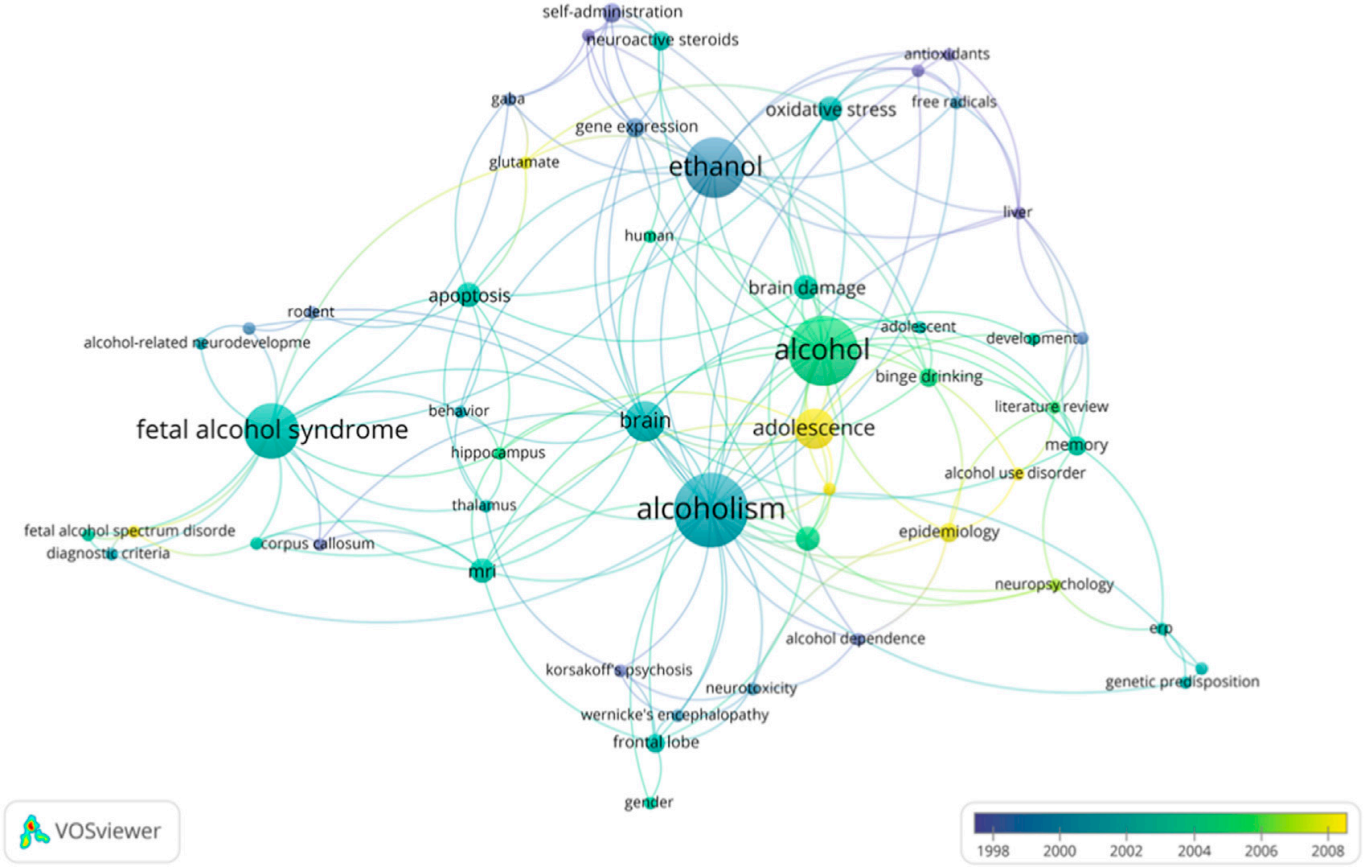
Overlay visualization of co-occurrence of author-keywords with a no minimum of two occurrences. Clusters are represented by different colors and the lines indicate co-citation links between keywords.

A total of 288 author keywords were part of the articles, with an occurrence variation of between 1 and 14 occurrences. The words most used by the authors were “alcoholism”, “alcohol”, and “ethanol”, used 14, 13, and 11 times respectively. These words also had the highest binding strengths among the keywords (Figure 6).

#### 3.1.5 Geographical distribution of corresponding authors of the top 100 articles

**FIGURE 7:**
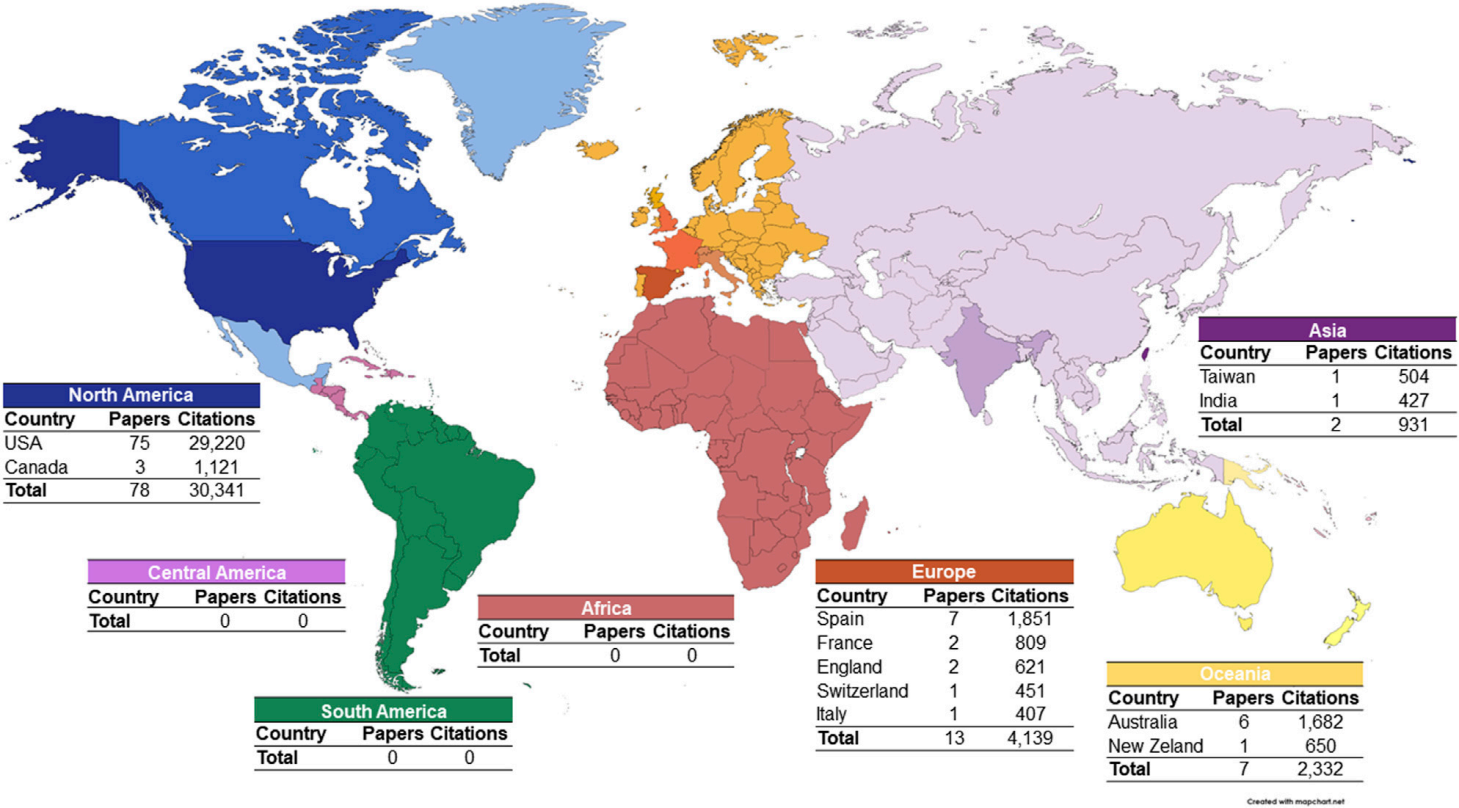
Geographical distribution of the corresponding authors of the 100 most cited articles on the neurotoxicology of alcohol.

The most relevant scientific production regarding the effects of alcohol on the nervous system is distributed across several countries; however, it is concentrated in the North American continent (n = 78 articles), more specifically in the United States, which contributed with 75 articles and 77.41% of the citations. The European continent contributed with 13 articles, a result mainly due to articles with corresponding authors from Spain (n = 7), England (n = 2), and France (n = 2). Africa, Central America, and South America did not present selected articles in this study (Figure 7).

### 3.2 Content analysis

#### 3.2.1 Study design

Among the 100 most cited articles, the most frequent study models were literature review (58%), *in vivo* laboratory study (17%), cohort (7%), and non-randomized clinical trial (7%). These study types received a total of 90.52% (34,165) of the total number of citations (Table 3).

**TABLE 3:** Study design of the 100 most-cited papers.

Study design	Number of articles	Number of citations WoS-CC^a^	Citation average
Literature review	58	23,795	410.26
*In vivo* laboratory study	17	5,841	343.59
Cohort	7	2,384	340.57
Non-randomized clinical trial	7	2,145	306.43
Practice guideline	4	1,454	363.50
Case-control study	3	960	320.00
*In vitro* laboratory study	2	476	238.00
Case series	1	455	455.00
Systematic review	1	234	234.00

^a^ WoS-CC, web of science core collection.

#### 3.2.2 Knowledge maps

In primary studies, several aspects were covered, such as mechanisms associated with alcohol-induced damage (He and Crews, 2008) and changes in the morphology (Caine et al., 1997) and function (Townshend and Duka, 2005) of different regions of the central nervous system. In addition, there were studies that addressed the prevalence of fetal alcohol syndrome (Clarren et al., 1997; May et al., 2018), updating of fetal alcohol syndrome diagnostic criteria (Astley and Clarren, 2000; Chudley et al., 2005; Hoyme et al., 2005; Hoyme et al., 2016), and improving the knowledge on Werneck encephalopathy criteria in people with alcohol use disorders (Caine et al., 1997).

Some studies evaluated these alterations in specific regions of the nervous system. Reductions in the cortes cortex (Kril et al., 1997) and corpus callosum volume (Riley et al., 1995; Bookstein et al., 2002) as well as the hippocampus region (De Bellis et al., 2000; Nagel et al., 2005) were explored. Furthermore, it was observed that women are more sensitive to these ethanol-induced damages (Hommer et al., 2001; Medina et al., 2008).

Among the experimental studies, doses ranging from 2.5 to 14 g/kg, with a dose of 5 g/kg being used more frequently (29.41% of the articles). These studies were carried out in different experimental models: in rats (Wistar and Sprague-Dawley) and mice (C56BL/7 and C57BL/6J); Sprague-Dawley rats were the most frequently used species (n = 9 articles, 52.94%). The majority of the selected articles addressed prenatal and postnatal exposure to alcohol, with postnatal exposure being the most frequently studied (n = 15 articles, 88.23%). Regarding the assessment of damage, most studies started the investigation in the first days of the animal’s life (Table 4).

**TABLE 4:** Knowledge mapping in experimental studies.

Author/Years	Animal	Dose	Exposure pathway	Exposure period	Time of exposure	Evaluated effect
Ikonomidou et al. (2000)	Rats Sprague-Dawley	5 g/kg/day	Subcutaneous	Prenatal (17th and 19th day) and Postnatal (0, 3, 7, 14 and 21th days of life)	1 day	Blockade of NMDA receptors and excessive activation of GABA receptors, promoting widespread apoptotic degeneration in the developing mouse forebrain
Pich et al. (1995)	Rats Wistar	*Ad libitum* (8.5% v/v)	Orally (*ad libitum*)	Postnatal (^a^)	2–3 weeks	Abstinence increased immunoreactivity to the corticotropin-releasing factor
Bonthius and West (1990)	Rats Sprague-Dawley	4.5 g/kg/day and 6.6 g/kg/day	Intragastric	Postnatal (4th days of life)	6 days	Neuronal loss, with worsening in chronic consumption
Crews et al. (2000)	Rats Sprague-Dawley	9–10 g/kg/day	Intragastric	Postnatal (33rd days of life)	4 days	Adolescent brain is more sensitive to alcohol-induced damage when compared to adult exposure
Alfonso-Loeches et al. (2010)	Rats Wistar (male) and mice C57BL/6 WT (female) and mice KO	12.8 ± 1.2 g/kg/day	Orally (*ad libitum*)	Postnatal (7th week of life)	5 months	Chronic alcohol intake increases microglial reactivity
Qin et al. (2008)	Mice C57BL/6J	5 g/kg/day (25% w/v)	Intraperitoneal	Postnatal (^a^)	1 and 10 days	Chronic alcohol exposure reduces proliferation of neuronal progenitor cells
Nixon and Crews (2002)	Rats Sprague-Dawley (male)	5 g/kg/day (30% w/v; 1 day)	Intragastric	Postnatal (^a^)	1 and 4 days	Chronic ethanol binge decreased proliferation of neuronal progenitor cells
		9.3 g/kg/day (25% w/v; 4 days)				
Roberto et al. (2003)	Rats Sprague-Dawley (male)	^a^	^a^	Tonsils collected and evaluated *in vitro* (^a^)	^a^	Increased GABAergic transmission in pre- and postsynaptic neurons in the central nucleus of the amygdala
Roberts et al. (1996)	Rats Sprague-Dawley (male)	^a^	Orally (*ad libitum*) e/or inhalation (EtOH vapor)	Postnatal (^a^)	^a^	Association of GABA receptors to dependence mechanisms
Pascual et al. (2007)	Rats Wistar	3 g/kg/day	Intraperitoneal	Postnatal (25th days of life)	2 weeks (2-day intermittent exposure and 2-day abstinence)	Intermittent ethanol administration during adolescence increased COX-2 and iNOS levels and neural cell death in several brain regions and caused long-lasting neurobehavioral impairments
VanDoren et al. (2000)	Rats Sprague-Dawley (male)	3.5 g/kg/day (20% v/v)	Intraperitoneal	Postnatal (^a^)	1 day	Ethanol, in moderate doses, induces a dose- and time-dependent increase in cerebral cortical 3α,5α-THP levels. This increase is associated with the behavioral and electrophysiological effects of ethanol
Roberts et al. (2000)	Mice C57BL/6	^a^	Orally (*ad libitum*) (5, 8.5% and 10% v/v)	Postnatal (5 and 6th month of life and 11th month of life animals in final phase)	40 days	Knockout mice with µ-opioid receptor showed aversion to alcohol
Olney et al. (2002)	Mice C57BL/6	2.5 g/kg/day	Subcutaneous	Postnatal (^a^)	1 day	Induces apoptotic neurodegeneration during synaptogenesis
Wozniak et al. (2004)	Mice C57BL/6	5 g/kg/day	Subcutaneous	Postnatal (60–90th days of life)	Acute effect: euthanized 8–24 h after treatment with alcohol	Exposure on the 7th day after birth induces intense apoptotic neurodegeneration, causing memory and learning impairments
					Chronic effect: P14, P30, and P90	
Harris et al. (1995)	Mice	0.02 ml/g of body mass/day (20% m/v)	Intraperitoneal	Postnatal (^a^)	^a^	Changes in GABA receptors abolished the effects of alcohol exposure
Livy et al. (2003)	Rats Sprague-Dawley (female)	6 g/kg (22.5% p/v) prenatal	Intragastric	Prenatal and postnatal (^a^)	^a^	Cell numbers were significantly reduced in the CA1 and CA3 regions of the hippocampus and in the dentate gyrus after exposure to alcohol during the equivalent third trimester
		5 g/kg (9.5% p/v)				
		Postnatal				
Herrera et al. (2003)	Rats Sprague-Dawley	6.4% v/v	^a^	Postnatal (^a^)	6 weeks	The effect of ethanol on the survival of newly formed neurons in the adult hippocampus may result in impairment of hippocampus-dependent cognitive functions

^a^ Not reported; v/v: weight/volume; v/v: volume/volume.

Among the observed results, the authors reported that alcohol consumption induces intense neuroinflammation with consequent apoptotic neurodegeneration, inducing alterations in several regions of the central nervous system. The hippocampus was the region of greatest interest, where it was shown that alcohol abuse induces a reduction in the population of neurons in this brain region which is associated with a decrease in learning and memory capacity.

## 4 Discussion

This study was based on the 100 most cited articles and focused on mapping knowledge about the neurotoxicology of alcohol. In the present study, it was observed that most of the articles were literature reviews and that they received a high average number of citations. The selected studies were carried out *in vitro*, in humans, or animals, with different modes of exposure to ethanol. In animals, exposure was evaluated in acute and chronic protocols, both with potentially dangerous doses, equivalent to heavy alcohol consumption (National Institute on Alcohol Abuse and Alcoholism, 2004). The main effects of ethanol neurotoxicity were evaluated especially during pregnancy and adolescence, when abusive consumption can lead to impaired neurodevelopment.

Several factors contribute differently to the outcome of ethanol exposure, among them the amount, frequency, and pattern of consumption (Molina and Nelson, 2018). The damages induced by ethanol consumption are proportional to the type and duration of exposure (Zakhari, 2006), so intense and episodic consumption has received special attention from several researchers due to this pattern of consumption providing high concentration of alcohol in the blood and this remain a longer period, given the limited ability to metabolize alcohol in determining time (Molina and Nelson, 2018; Simon et al., 2022). In this perspective, the studies selected for this mapping made use of an alcohol exposure model that mimics exposure in binge drinking in humans, making it clear that scientists are concerned with this exposure model, given the risk of this form of exposure to induce damage. Biochemical, molecular, and tissue effects in part and/or throughout the nervous system.

Excessive alcohol consumption induces neurodegeneration and cortical dysfunction, this dysfunction associated with impulsivity and contribute to the consumption of dangerous amounts of alcohol. In addition, frequent consumption of high doses of alcohol induces neuroadaptive processes that lead to tolerance, dependence, and manifestations of withdrawal syndrome (Fadda and Rossetti, 1998; Crews and Nixon, 2009).

Considering the historical perspective of ethanol neurotoxicology, this analysis identified and selected studies considered “classic”, which influenced the knowledge of this topic and thus contributed to the development of new researches (Ahmad et al., 2020). The number of citations was considered as an indicator of the quality and impact of a scientific work (Ahmad et al., 2020). Among these articles, the most cited article reviewed the critical period of neurodevelopment, investigating the impact of alcohol exposure during different periods in humans and rats (Rice and Barone, 2000).

The WoS-CC was used as a reference database for using citation metrics from 1945, while a comparison was performed with the Scopus database, whose metrics date from 1966, and Google Scholar. Google citation values were higher than citations in WoS-CC and Scopus, since the Google database counts citations in articles, books, congress annals, academic works (dissertations and theses), reports, and pre-prints differently from the WoS-CC and Scopus platforms. Thus, the difference in number of citations between the databases for the same article is due to the exclusive method of recording and counting citations in each database (Bakkalbasi et al., 2006; Kulkarni et al., 2009).

The three journals that published the most were the journals “Alcoholim-clinical and experimental research”, “alcohol and alcoholis” and “Alcohol research and health”, specific journals on ethanol, not appearing among the journals that most published any public health journal and/or collective health, showing that much of the most cited production is centred on biological and behavioral aspects of the effects of ethanol.

The most cited author was Koob GF, with 3513 citations and five articles. This researcher is recognized worldwide for dedicating his career to the study of alcohol and the neurobiology of alcohol and other drugs. He is currently Director of the NIAAA, which investigates several aspects of alcohol research, ranging from basic science to diagnosis, prevention, treatment, and epidemiology. Koob’s articles are among the 100 most cited, addressing important aspects of ethanol neurotoxicology, such as mechanisms involved in alcohol dependence, including reward pathways involved in this abuse process, in addition to the evaluation of therapeutic alternatives for the treatment of problems related to alcohol.

When evaluating the geographic issue and the production of knowledge by country, it is important to consider that alcohol consumption has become a social problem with an impact on public health in several countries (Harper, 2009; Petrella et al., 2020), among them the United States, where alcohol is the most consumed intoxicant among adolescents (Medina et al., 2008). It is estimated that 19% of adolescents and 7% of adults have some degree of dependence or excessive consumption, and such abusive use has been found to be one of the leading causes of death in the United States (Esser et al., 2020), responsible for 10% of premature deaths in working-age adults (Stahre et al., 2014). These data justify the North American community’s great interest in studying alcohol abuse and its effects, especially on the central nervous system. Our results showed that 75% of the selected articles are by authors affiliated with North American institutions and 60% of the articles state that they received funding from the National Institutes of Health (NIH).

Among the 37 selected primary studies, 20 assessed the effects of alcohol through prenatal exposure. Prenatal exposure studies are of great relevance, especially in the initial period of neurodevelopment, allowing a better understanding of the mechanisms involved in changes in this process, since the effects of alcohol are associated with its ability to cross the blood-brain barrier. In this sense, alcohol can induce damage to the developing nervous system by several mechanisms.

Prenatal exposure occurs at a critical period in neurodevelopment, leading to changes in proliferation, migration, differentiation, synaptogenesis, myelination, and apoptosis (Rice and Barone, 2000). Prenatal alcohol exposure results in learning, memory, and attention deficits due to the teratogenic effect of alcohol, which causes abnormal morphofunctional development of the hippocampus (Berman and Hannigan, 2000); *in vitro* and *in vivo* studies have demonstrated abnormal projections of axons and reduction of pyramidal neurons in the hippocampus as a result of prenatal alcohol exposure (Berman and Hannigan, 2000). Furthermore, an association with depressive and psychotic disorders in adulthood has been reported (Olney et al., 2006).

During embryogenesis, glial cells play a critical role; radial glia provide guidance for the migration of neurons, in addition to acting as a neural precursor cell with potential for self-renewal and generation of neurons and oligodendrocytes (Guerri et al., 2009). In addition, synaptogenesis is a critical period in synaptic function, where glial cells promote synapse formation and regulate neurotransmitters and energy in the developing brain (Guerri et al., 2009). Alterations in these mechanisms, as occur in ethanol exposure, can induce irreversible damage to the nervous system (Guerri et al., 2009).

Postnatal exposure was evaluated by 17 articles, in which it was observed that the effects are directly proportional to the type and period of exposure: the earlier the exposure to ethanol, the greater the intensity of damage (Eckardt et al., 1998; Nixon and Crews, 2002; Goodlett et al., 2005; Guerri et al., 2009; Thompson et al., 2009). Among human studies, exposure to alcohol in adolescence has received particular attention (n = 8 papers). Adolescence is considered a period sensitive to the neurotoxic effects of alcohol use (Medina et al., 2008). This period is marked by the development of the prefrontal cortex, structures of the limbic system, and white matter association fibers (Bava and Tapert, 2010). These processes are associated with the development of more sophisticated cognitive functions and emotional processing (Bava and Tapert, 2010).

Excessive alcohol consumption (binge drinking or heavy alcohol use) is prevalent and severe in this period and can lead to changes in the development of these structures (De Bellis et al., 2000; Bava and Tapert, 2010), and the frequent consumption of high doses of alcohol induces neuro-adaptive processes that lead to tolerance, dependence, and manifestations of the withdrawal syndrome (Fadda and Rossetti, 1998; Paus et al., 2008; Crews and Nixon, 2009).

The selected studies showed differences between genders, with women being more sensitive and more vulnerable to the hazardous effects induced by chronic exposure to alcohol (Mumenthaler et al., 1999; Hommer et al., 2001; Medina et al., 2008; Courtney and Polich, 2009). Hommer et al. (2001) and Medina et al. (2008) demonstrated that women addicted to alcohol had smaller volumes of white and gray matter than non-dependent women, and these differences were smaller when the same groups were compared among men. These results may be related to the difference in blood glucose levels and levels of aldehyde dehydrogenase between genders, in addition to the fact that women reach higher concentrations of alcohol with alcohol consumption in a similar dose to men (Mumenthaler et al., 1999).

Several mechanisms, acting synergistically and in different regions, have been proposed to explain brain damage induced by alcohol exposure (Harper and Matsumoto, 2005). Selected studies assessed injury to the hippocampus (n = 6 papers), cortex (n = 5 papers), corpus callosum (n = 2 papers), amygdala (n = 1 papers), and thalamic nucleus (n = 1 papers).

The nervous system is characterized by high metabolic activity with high oxygen consumption and has a high content of polyunsaturated fatty acids and catecholamines, both of which are easily oxidizable. Neuronal death induced by alcohol consumption is associated with increased oxidative stress and expression of pro-inflammatory proteins (Crews and Nixon, 2009). The oxidation of alcohol by the activity of catalase and alcohol dehydrogenase leads to the formation of free radicals (Nordmann et al., 1992). In this context, Pascual et al. (2007) demonstrated increases in cyclooxygenage-2 (COX-2) and nitric oxide synthase (iNOS) and consequent neuronal death in an animal model.

Chronic alcohol consumption leads to regulation of N-methyl-D-aspartate (NMDA) receptors, as well as excitatory amino acid receptors (De Bellis et al., 2000). During abstinence, there is an increase in excitatory transmission, which can lead to excitotoxicity due to the increased stimulation of these receptors (De Bellis et al., 2000). Alcohol modulates several neurochemical systems or their relationship, among them the GABA, glutamatergic, serotonergic, dopaminergic, and opioid neuronal systems (Eckardt et al., 1998).

During the mapping of the most cited articles, a search to understand the mechanisms of addiction and dependence involved in alcohol drug addiction was observed. In this sense, Roberts *el al.* (1996) (Roberts et al., 1996) and Roberto et al. (2003) evaluated neuropharmacological mechanisms (GABA and glutamate) related to the reinforcement and pathways of neurotransmitters involved in reward in alcohol addiction and thus opening the possibility of investigations into new pharmacological therapies and advances in research. These studies reinforce ethanol–GABA interaction in the central nucleus of the amygdala (Roberts et al., 1996; Roberto et al., 2003). In addition, the cannabinoid system is distributed throughout the nervous system and is associated with functions of motor control, cognition, emotion, and motivation. In this context, de de Fonseca et al. (2005) associated the endogenous cannabinoid system with drug addiction to alcohol, suggesting the use of a cannabinoid receptor antagonist as a therapeutic alternative in dependence, and relapse related to alcohol abuse.

Among the selected articles, the effects of alcohol on the prefrontal cortex (Moselhy et al., 2001; Medina et al., 2008; Crews and Boettiger, 2009), hippocampus (Berman and Hannigan, 2000; De Bellis et al., 2000; Nixon and Crews, 2002; Livy et al., 2003), cerebellum (da Silva et al., 2018; Lamarão-Vieira et al., 2019), thalamic nucleus (Harding et al., 2000), amygdala (Pich et al., 1995; Roberto et al., 2003), and corpus callosum (Riley et al., 1995; Bookstein et al., 2002). However, the mechanisms of alcohol neurotoxicity are still not completely understood, requiring studies that evaluate other regions of the nervous system, such as the spinal cord, where initial studies show sensitivity to alcohol (da Silva et al., 2018) as well as to other toxic substances (da Silva et al., 2022; Eiró-Quirino et al., 2023).

The bibliometric study is based on the number of citations and, therefore, allowing to identify the “hot topics”, dynamic trend and current trends on a topic (Jani et al., 2020; Yanbing et al., 2020), is one of the limitations of this type of study, since it is a purely quantitative evaluation. The limitation of the present assessment relies on the absence of analysis of the quality of the selected study or scientific method nor the certainty of evidence of these articles. In addition, more recent and robust articles may be outside the classification of the selected list, since there has not been enough time to accumulate the number of citations and appear among the 100 most cited articles. The results presented here do not allow for decision-making in public and/or collective health, but which still bring important elucidations showing the need for well-designed epidemiological studies with greater dissemination in the scientific community. There are gaps regarding studies with more evidence and it is relevant for researchers to pay attention to the need for clinical studies, the growing consumption of alcohol by women and young people, new patterns of consumption, such as heavy alcohol use, and the consequences of short- and long-term abstinence.

## 5 Conclusion

This study identified and analyzed the 100 most cited articles on the neurotoxicology of alcohol using the citation analysis method; most articles were selected from literature reviews and had corresponding authors affiliated with North American institutions. The articles assess the effects of pre- and postnatal alcohol exposure. Prenatal exposure presents data of great interest regarding the risk of experiencing fetal alcohol syndrome, while postnatal exposure, especially in adolescence, is associated with cognitive and memory impairment.

## Data Availability

The original contributions presented in the study are included in the article/Supplementary material, further inquiries can be directed to the corresponding author.
